# Gastric cancer simultaneously complicated with extrahepatic bile duct metastasis and portal vein tumor thrombus: a case report

**DOI:** 10.1186/s40792-023-01764-y

**Published:** 2023-10-17

**Authors:** Naohiko Otsuka, Yasuhiko Nakagawa, Hiroshi Uchinami, Yuzo Yamamoto, Junichi Arita

**Affiliations:** Department of Gastroenterological Surgery, Akita University Graduate School of Medicine, 1-1-1, Hondo, Akita, 010-8543 Japan

**Keywords:** Extrahepatic bile duct metastasis, Gastric cancer, Portal vein tumor thrombus

## Abstract

**Background:**

Gastric cancer metastatic to the extrahepatic bile duct or accompanied by portal vein tumor thrombus (PVTT) is rare. To our knowledge, there have been no cases complicated with both of these factors.

**Case presentation:**

A 72-year-old man presented with icterus and melena. A biochemical blood test showed abnormal values for hepatobiliary enzymes and a tumor marker, and abdominal computed tomography scan revealed wall thickening of the lower bile duct with intra- and extra-hepatic bile duct dilatation and PVTT. A biopsy of the lower bile duct during endoscopic retrograde cholangiopancreatography demonstrated a moderately differentiated tubular adenocarcinoma. Moreover, gastroduodenoscopy showed a type 3 tumor at the lesser curvature of the gastric antrum, and an endoscopic biopsy demonstrated a moderately differentiated tubular adenocarcinoma. We diagnosed concomitant gastric cancer and distal bile duct accompanied by PVTT, and pancreatoduodenectomy with combined resection of the portal vein was performed. The resected specimen revealed a tumor in the lesser curvature of the gastric antrum and circumferential wall thickening in the lower bile duct. In pathological findings, infiltration of a moderately differentiated tubular adenocarcinoma from the mucosal layer to the subserosal layer of the stomach was observed. In contrast, a moderately differentiated tubular adenocarcinoma demonstrating the same histological type as the gastric cancer had spread not to the mucosal layer but mainly to the fibromuscular layer of the lower bile duct. Immunohistochemical staining showed identical patterns between gastric cancer and the bile duct tumor: negativity for cytokeratin 7 (CK7), and positivity for CK19 and 20. Therefore, the final diagnosis was extrahepatic bile duct metastasis from gastric cancer with PVTT. Unfortunately, multiple liver metastases occurred in the early postoperative period and chemotherapy was conducted, but the patient died 12 months after the surgery.

**Conclusions:**

In the diagnosis of extrahepatic bile duct metastasis, immunohistochemical staining of gastric cancer and the bile duct tumor was essential and helpful as decisive evidence.

## Background

Metastasis of the malignant tumors to the extrahepatic bile duct is rare, and even less common in patients with gastric cancer [1, 2]. In addition, gastric cancer is seldom accompanied by portal vein tumor thrombus (PVTT), at a rate of approximately 1% [3, 4]. Herein, we report an extremely rare case of gastric cancer simultaneously complicated with extrahepatic bile duct metastasis and PVTT.

## Case presentation

**Fig. 1:**
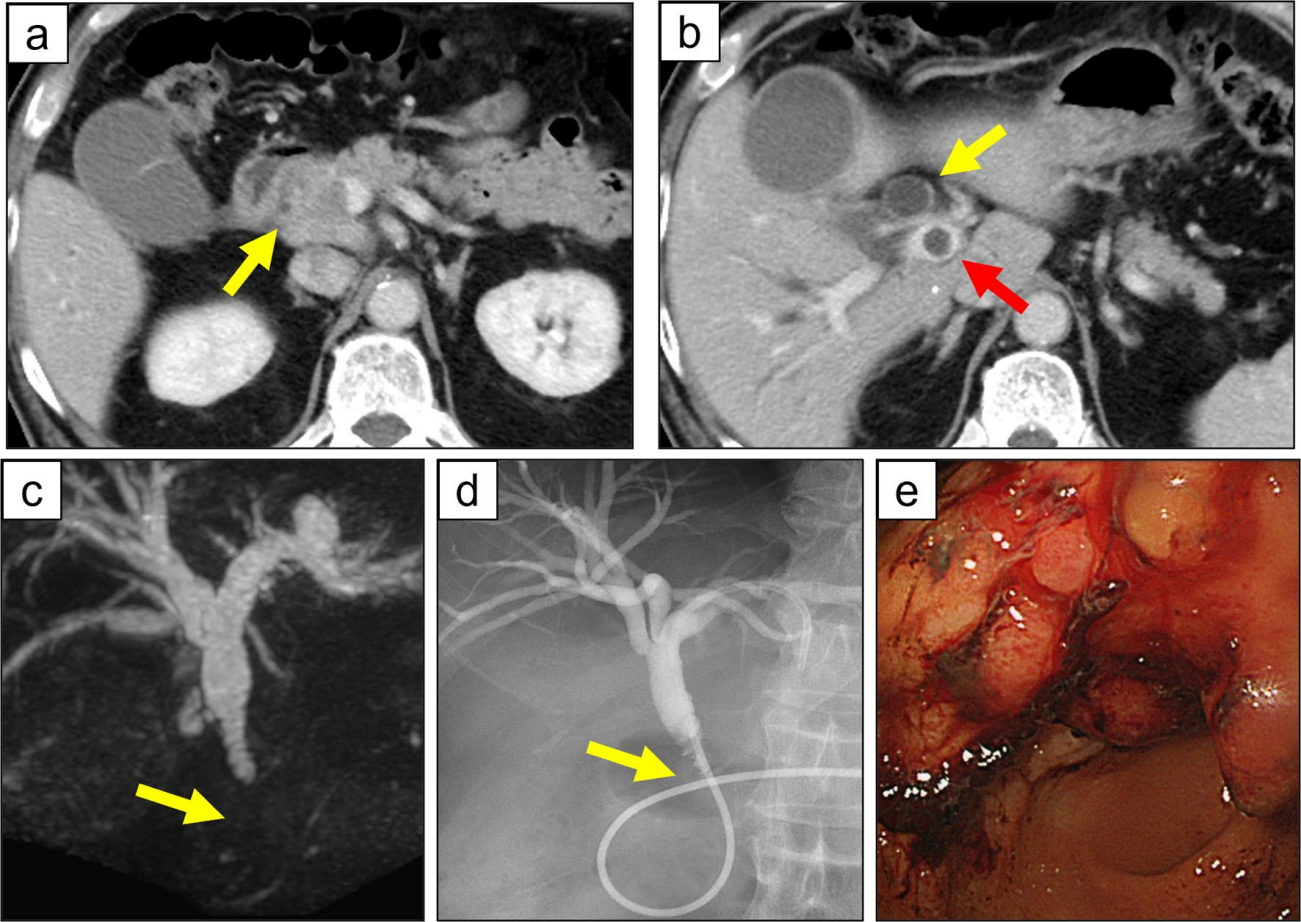
Imaging and endoscopic findings. **a** Abdominal computed tomography (CT) scan showed wall thickening of the lower bile duct (arrow). **b** Proximal side of the thickened bile duct was dilated (yellow arrow), and low density area in the portal vein on contrast-enhanced CT scan was considered a tumor thrombus (red arrow). **c** A defect in the lower bile duct (arrow) and dilatation of the upper side were revealed on magnetic resonance cholangiopancreatography. **d** Cholangiography showed the same findings as magnetic resonance cholangiopancreatography. The arrow indicated a defect in the lower bile duct. **e** Gastroduodenoscopy showed a type 3 tumor at the lesser curvature of the gastric antrum

A 72-year-old man presented with icterus and melena and was referred to our hospital. A biochemical blood test showed abnormal values for hepatobiliary enzymes (total bilirubin, 18.0 mg/dL; aspartate aminotransferase, 310 IU/L; alanine aminotransferase, 417 IU/L; alkaline phosphatase, 1958 IU/L; gamma-glutamyl transpeptidase 1141 IU/L) and carbohydrate antigen 19–9 had increased to 168 U/mL. Abdominal computed tomography (CT) scan showed wall thickening of the lower bile duct with dilatation of intra- and extra-hepatic bile ducts and PVTT (Fig. 1a, b). Magnetic resonance cholangiopancreatography revealed a defect in the lower bile duct and dilatation of intra- and extra-hepatic bile ducts (Fig. 1c). Endoscopic retrograde cholangiopancreatography was performed (Fig. 1d) and a biopsy of the lower bile duct demonstrated a moderately differentiated tubular adenocarcinoma. Furthermore, gastroduodenoscopy demonstrated a type 3 tumor at the lesser curvature of the gastric antrum (Fig. 1e), and an endoscopic biopsy demonstrated a moderately differentiated tubular adenocarcinoma. Because abdominal CT scan did not demonstrate swollen lymph nodes compressing the bile duct and gastric cancer was not adjacent to the bile duct tumor, we diagnosed concomitant primary cancer in the stomach and the bile duct accompanied by PVTT.

**Fig. 2:**
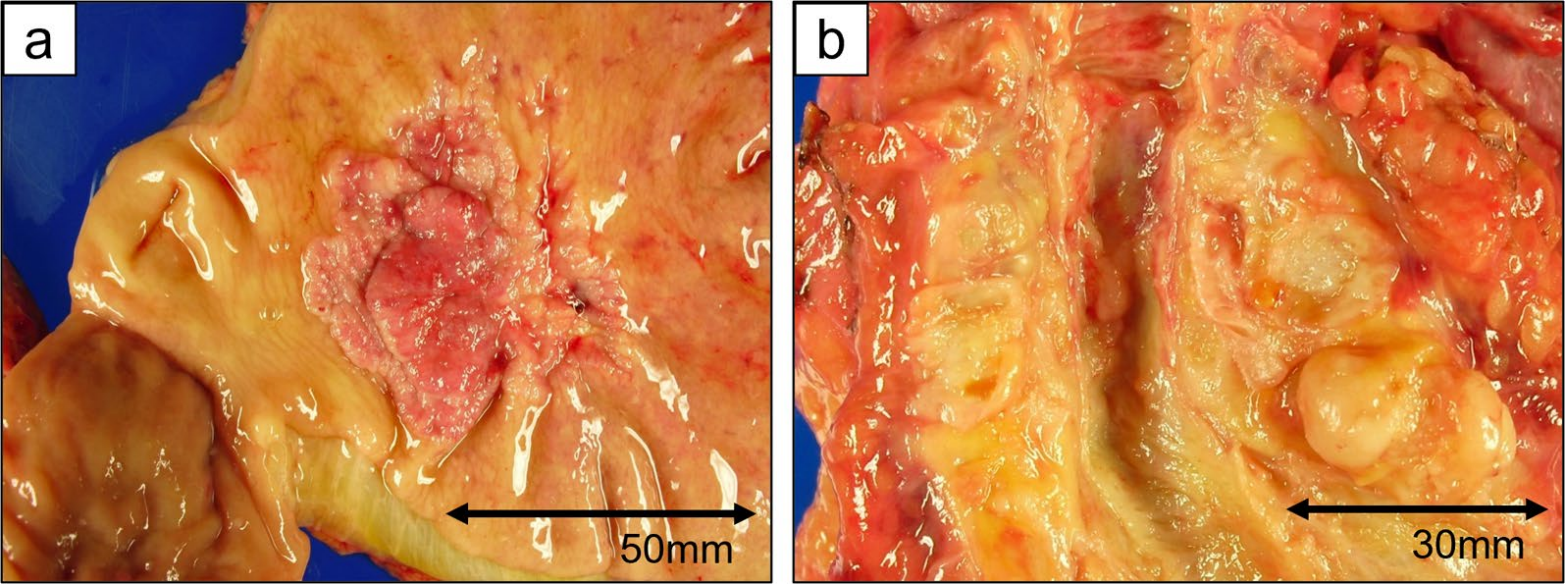
Macroscopic findings of the resected specimen. **a** A type 3 tumor measuring 50 × 50 mm was located in the lesser curvature of the gastric antrum. The tumor did not expose to the serosal surface. **b** The wall of the lower bile duct was being thickened circumferentially, 35 mm in length

**Fig. 3:**
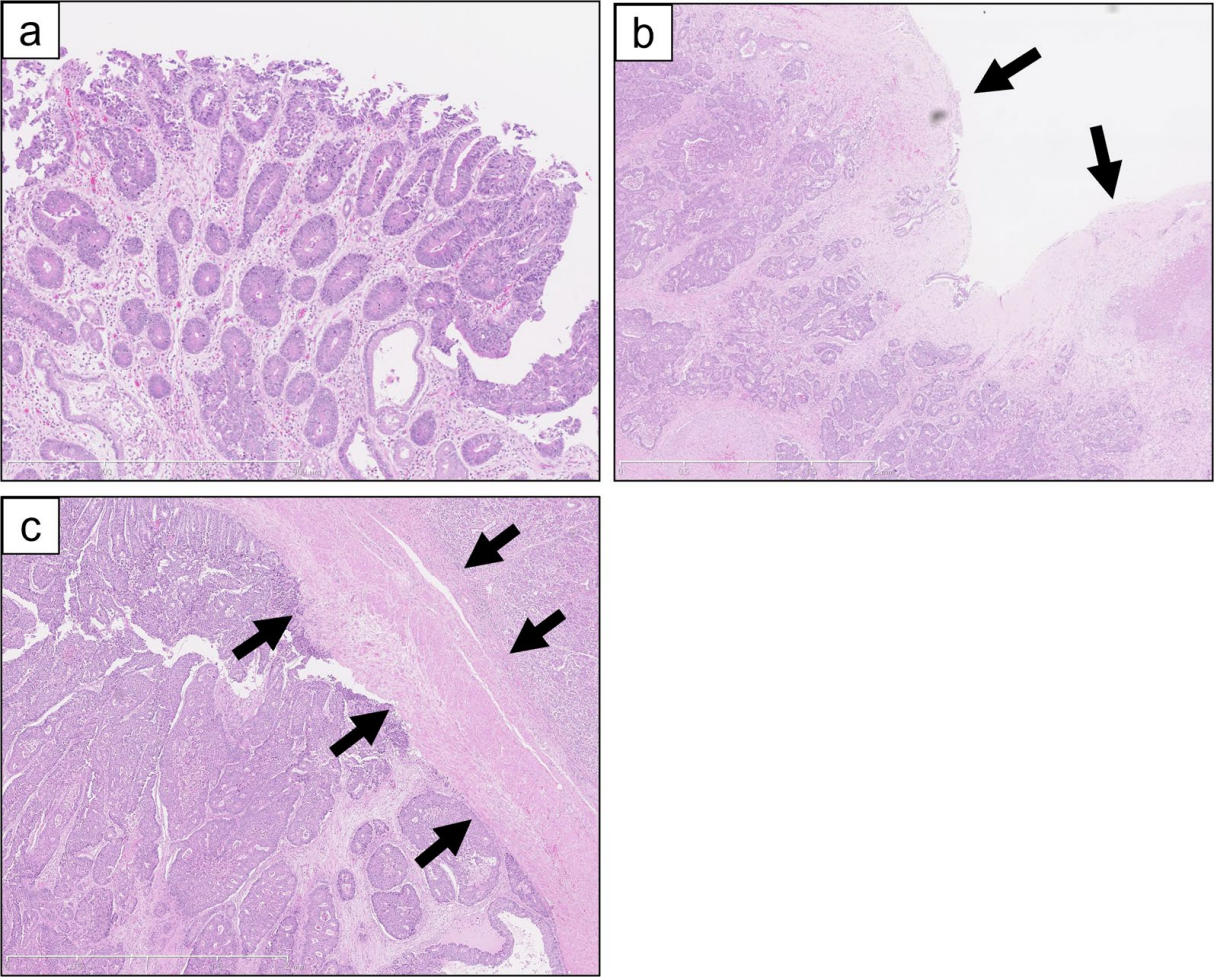
Histopathological findings. **a** Histological section of gastric tumor demonstrated a moderately differentiated tubular adenocarcinoma with infiltration from the mucosal layer to the subserosal layer (hematoxylin and eosin staining; × 100). **b** There were no cancer cells in the mucosa of the lower bile duct (arrows). A moderately differentiated tubular adenocarcinoma demonstrating the same histological type as the gastric cancer had spread mainly to the fibromuscular layer (hematoxylin and eosin staining; × 20). **c** Cancer cells infiltrated the portal vein, forming a tumor thrombus. Arrows show the wall of the portal vein (hematoxylin and eosin staining; × 20). The tumor thrombus is to the left of the portal vein wall

**Fig. 4:**
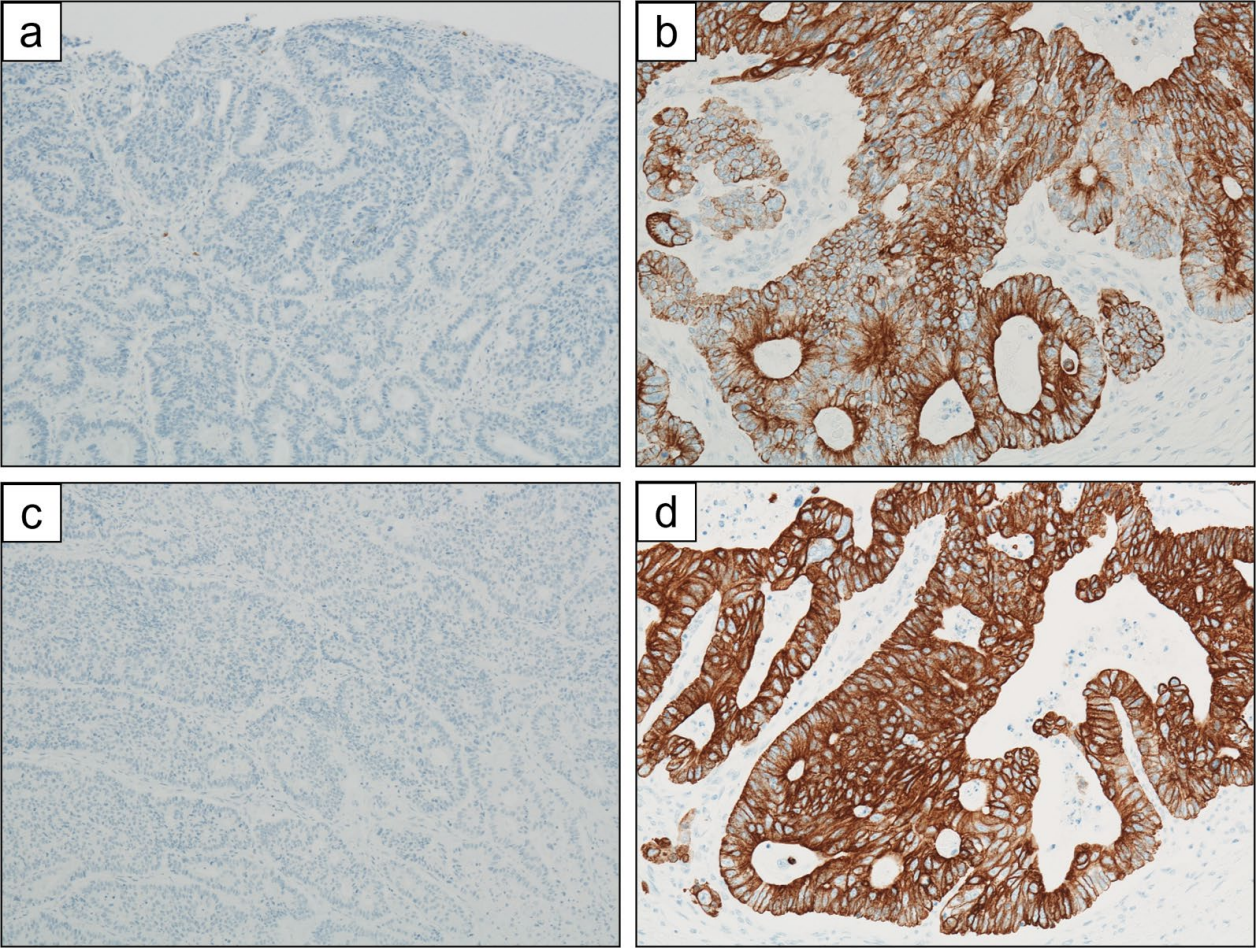
Immunohistochemical staining. Gastric cancer was negative for cytokeratin 7 (CK7) (**a**; × 100), and positive for CK20 (**b**; × 200). The results of the bile duct tumor were consistent with those of gastric cancer (**c**; × 100, **d**; × 200)

Pancreatoduodenectomy with combined resection of the portal vein was planned. After laparotomy, liver or peritoneal metastasis was not found, and cancer cells were not detected in peritoneal cytology. Thus, we proceeded with the surgery as planned. Since gastric cancer was located at the lesser curvature of the gastric antrum based on gastroduodenoscopy, we secured a sufficient margin using a classical pancreatoduodenectomy procedure, where the stomach was divided at the border between right and left gastroepiploic arteries. Lymph nodes around the left gastric artery, celiac artery and splenic artery, and along the lesser curvature of the stomach were dissected in addition to standard lymphadenectomy through pancreatoduodenectomy. Combined resection of the portal vein to remove the PVTT was performed with clamping just upstream of the bifurcation and just downstream of the spleno-portal junction. A 4-cm right external iliac venous graft was excised and used to interpose the stumps of the portal vein. The resected specimen revealed a tumor measuring 50 × 50 mm in the lesser curvature of the gastric antrum and circumferential wall thickening in the lower bile duct measuring 35 mm in length (Fig. 2). Pathological examination revealed infiltration of a moderately differentiated tubular adenocarcinoma from the mucosal layer to the subserosal layer of the stomach (Fig. 3a) and showed advanced lymphatic and venous infiltration. In contrast, no cancer cells were found in the mucosa of the lower bile duct, while a moderately differentiated tubular adenocarcinoma demonstrating the same histological type as the gastric cancer had spread mainly to the fibromuscular layer (Fig. 3b). Immunohistochemical staining showed identical patterns between gastric cancer and the bile duct tumor: negativity for cytokeratin 7 (CK7), and positivity for CK19 and 20 (Fig. 4). Moreover, cancer cells infiltrated the portal vein, forming a tumor thrombus (Fig. 3c). Based on the above findings, the final diagnosis was extrahepatic bile duct metastasis from gastric cancer with PVTT (T3, N3a, M1 (extrahepatic bile duct), Stage IV, according to the 8th Edition of the TNM Classification of Malignant Tumours by Union for International Cancer Control). Gastric cancer showed HER2-negative (score 2 + in immunohistochemical staining and HER2/CEP17 < 2.0 in fluorescence in situ hybridization).

The postoperative course was uneventful without pancreatic fistula. However, abdominal CT scan on postoperative day 48 revealed multiple metastases in the whole liver. Chemotherapy using low-dose fluorouracil plus cisplatin instead of the recommended first line regimen for HER2-negative gastric cancer such as S-1 plus cisplatin and capecitabine plus oxaliplatin was commenced because we had to reduce the treatment intensity due to deterioration of general condition after the surgery, but unfortunately lung metastases emerged in addition to liver metastases, resulting in the death of the patient 12 months after the surgery.

## Discussion

Gastric cancer tends to metastasize to the lymph nodes, liver, and peritoneum [5, 6]. In contrast, recurrent tumors that cause biliary obstruction are reportedly seen in 1.4% to 2.3% of patients undergoing resection of gastric cancer, and in most of these cases, the recurrence sites are the lymph nodes in the hepatoduodenal ligament. Thus, cases of isolated extrahepatic bile duct metastasis from gastric cancer are rare [7, 8]. A search of the literature from 1980 to the present day using Igaku Chuo Zasshi, a web-based Japanese literature engine, and PubMed (keywords: gastric cancer, bile duct metastasis) revealed only four cases [9–12]. We summarized all five known cases including the present case (Table 1). Only Poletto et al. reached a diagnosis of extrahepatic bile duct metastasis from gastric cancer before treatment. In their report, immunohistochemical staining showed the markers expressed in the biopsied tissue from the bile duct and the primary site in the stomach were the same. The case described by Poletto et al. and the present case were similar in that the biopsy from the bile duct demonstrated cancer. It is paradoxical because the metastatic site in the bile duct mainly spread to the fibromuscular layer, and not to the mucosal layer, in all five cases. As Poletto et al. suspected, biopsy tissue might be obtained concomitantly from the mucosal surface and the fibromuscular layer. Similar to previous reports, we diagnosed extrahepatic bile duct metastasis from gastric cancer on the basis of the following: absence of cancer cells in the mucosa of the bile duct; the same histological type between gastric cancer and the bile duct tumor; and the same findings of immunohistochemical staining. In particular, immunohistochemical staining is critical as decisive diagnostic evidence.

**Table 1:** Summary of the five cases of extrahepatic bile duct metastasis from gastric cancer

Author	Age/sex	Period of metastasis	Diagnosis	Treatment	Metastatic site	Outcome
Yamamoto [9] (2008)	69 / M	28 months after gastrectomy	Lymph node metastasis in the hepatoduodenal ligament	Bile duct resection	Middle bile duct	Died 22 months later
Satake [10] (2013)	56 / M	Simultaneous	Distal bile duct cancer	ERBD	Lower bile duct	Died 2 months later
Takaichi [11] (2018)	66 / M	84 months after gastrectomy	Perihilar bile duct cancer	Extended left hepatectomy	Hilar bile duct	No recurrence for 6 months
Poletto [12] (2022)	56 / M	84 months after gastrectomy	Extrahepatic bile duct metastasis from gastric cancer	PD	Lower bile duct	No recurrence for 6 months
Present case (2023)	72 / M	Simultaneous	Distal bile duct cancer	PD	Lower bile duct	Died 12 months later

*ERBD* endoscopic retrograde biliary drainage, *PD* pancreatoduodenectomy

The present case is, to our knowledge, the first to report gastric cancer simultaneously complicated with extrahepatic bile duct metastasis and PVTT. Ozeki et al. mentioned the three developmental mechanisms of PVTT accompanied by gastric cancer: formation of tumor thrombus due to direct infiltration to the portal vein; infiltration from liver metastasis to the portal vein; and coexistence of gastric cancer and hepatocellular carcinoma complicated with PVTT [13]. Because the present case showed neither liver metastasis nor coexistent hepatocellular carcinoma, the gastric cancer directly infiltrated the portal vein.

In the present case, it was very difficult to identify the metastatic route because of the presence of many lymph node metastases and PVTT. Although gastric cancer showed advanced lymphatic involvement in the supra-pyloric site and hepatoduodenal ligament neighboring the lower bile duct, which may indicate metastatic route from gastric cancer to the bile duct via a lymphatic route, we considered the metastatic route via a hematogenous route was more likely based on the following findings. Firstly, advanced histological venous involvement was observed in the primary gastric site. Secondly, cancer cells slightly infiltrated the pancreatic tissues between PVTT and the site of bile duct metastasis. Thirdly, PVTT directly indicated hematogenous infiltration. Fourthly, multiple liver metastases followed by lung metastases, both of which were supposed to develop hematogenously, were seen in the early postoperative period.

As a treatment strategy, systemic chemotherapy is generally recommended for gastric cancer with distant metastasis or PVTT. Whereas it is difficult to discuss the treatment strategy for extrahepatic bile duct cancer with PVTT because few cases have been reported previously [14, 15], some papers reported long-term survival after resection of gastric cancer with PVTT following systemic chemotherapy [16, 17]. Waseda et al. reported that chemotherapy should be conducted at first even if gastric cancer with PVTT is considered resectable because PVTT is a poor prognostic factor [18]. Although we preoperatively diagnosed simultaneous gastric cancer and distal bile duct cancer, we could have diagnosed that the bile duct tumor was a metastatic lesion and that PVTT was derived from gastric cancer if immunohistochemical staining for biopsied tissues had been performed before the treatment. If we had reached the correct diagnosis, we would have selected systemic chemotherapy for far-advanced gastric cancer.

## Conclusion

We reported the first case of gastric cancer simultaneously complicated with extrahepatic bile duct metastasis and PVTT. In cases of bile duct lesion accompanied by malignant tumors such as gastric cancer, immunohistochemical staining is helpful in making a correct diagnosis.

## Data Availability

All data are available within this article.

## Abbreviation

CK: Cytokeratin
CT: Computed tomography
PVTT: Portal vein tumor thrombus
